# A Comprehensive Review of the Pathophysiology and Management of Osteoarthritis: Emerging Therapeutic Strategies and Clinical Insights

**DOI:** 10.7759/cureus.106561

**Published:** 2026-04-07

**Authors:** Abhay Harsh Kerketta, Arnav Pankaj Rathod, Junaid Nagori, Priyanka R Bhagat, Jyoti Chakraborty, Kadiervel K

**Affiliations:** 1 Orthopedics, Netaji Subash Medical College and Hospital, Jamshedpur, IND; 2 Orthopedics, Dr. Panjabrao Deshmukh Memorial Medical College and Hospital, Amravati, IND; 3 Orthopedics, Hamdard Institute of Medical Sciences and Research, New Delhi, IND; 4 Otorhinolaryngology, Dr. N. D. Desai Faculty of Medical Science and Research, Dharmsinh Desai University, Nadiad, IND; 5 Physiotherapy, School of Physiotherapy, Vinayaka Mission's Research Foundation (Deemed to Be University), Puducherry, IND

**Keywords:** diagnostics, osteoarthritis, regenerative therapies, structural degeneration, therapeutic strategies

## Abstract

Osteoarthritis persists as a major contributor to chronic joint impairment, driven by progressive disruption of cartilage integrity, synovial activity, subchondral bone dynamics, and biomechanical stability. Its multifactorial nature links metabolic influences, inflammatory mediators, and mechanical loading patterns to the diverse spectrum of clinical manifestations and structural outcomes observed across affected individuals. This synthesis integrates current understanding of disease mechanisms with evaluation of established and emerging management strategies designed to support joint function and symptom control. Evidence was gathered through focused examination of literature published between 2015 and 2026, emphasizing diagnostic advances, pharmacologic and non-pharmacologic measures, surgical interventions, and regenerative modalities used in clinical settings. Key insights highlight evolving recognition of early molecular alterations, improved imaging sensitivity, and expanding interest in biological approaches capable of influencing cellular pathways involved in joint deterioration. Conventional therapies continue to offer substantial symptomatic benefit, although structural modification remains limited despite ongoing therapeutic exploration. Regenerative strategies, targeted molecular agents, and refined surgical techniques present promising avenues, yet inconsistent response patterns and limited long-term data indicate ongoing complexity in optimizing care. Overall, current findings illustrate notable progress in understanding OA progression and support continued refinement of diagnostic frameworks and treatment pathways to enhance precision and effectiveness across varied clinical presentations observed globally.

## Introduction and background

Osteoarthritis (OA) is an important chronic disabling condition impacting millions of people across the world's population and exerting growing burdens on medical care systems [1]. Joints are structures where two bones meet, and articular cartilage is a smooth, cushioning tissue that reduces friction and absorbs mechanical load during movement. In simple terms, cartilage functions like a shock absorber between bones [2]. Gradual loss of joint structures is accompanied by chronic pain and reduced mobility, leading to a measurable decline in functional ability and overall quality of life [3]. Previously, OA was described primarily as an erosion of hyaline cartilage. However, more recent evidence portrays it as a multifaceted disease process involving integrated changes in multiple joint components, including cartilage, the synovium (the soft tissue lining the joint), subchondral bone (the layer of bone beneath cartilage), menisci, periarticular muscles, and supporting ligaments [4].

Mechanical overload, persistent low-grade inflammation, metabolic dysregulation, and cellular senescence contribute to disease progression [5]. These processes interact through overlapping biological pathways and result in different clinical presentations, often referred to as disease phenotypes (distinct patterns of disease expression) [6]. At a broader level, these interacting mechanisms form the basis of structural joint degeneration, while at a cellular level, they drive molecular alterations such as extracellular matrix (ECM) degradation and inflammatory mediator release [7,8]. This broader understanding highlights the complexity of OA and emphasizes the limitations of treatment strategies that focus on a single tissue or mechanism. Increasing life expectancy, rising obesity rates, and occupational stress contribute to the growing incidence and severity of OA worldwide [9,10]. The development of symptoms is often preceded by years of silent structural changes within the joint, contributing to delayed clinical diagnosis [11].

Early detection and diagnostic limitations

Radiography remains widely used for diagnosis. However, it has a limited ability to detect early biochemical or microstructural changes in joint tissues [12]. As a result, OA is often identified only at advanced stages of joint damage. This reduces the opportunity for early intervention and limits the ability to alter disease progression. Advanced imaging modalities, including magnetic resonance imaging (MRI) and emerging radiomics-based approaches, have shown potential in identifying early structural and compositional changes, although their routine clinical application remains limited. Additionally, available biomarkers lack sufficient validation for routine clinical use [13].

Current management strategies

Current management strategies primarily focus on symptom relief and maintenance of joint function [2]. Pharmacological treatments include nonsteroidal anti-inflammatory drugs (NSAIDs), corticosteroid injections, and hyaluronic acid injections. These interventions can reduce pain but have minimal impact on the underlying disease mechanisms [4].

Non-pharmacological approaches, such as exercise therapy, weight management, physiotherapy, and orthotic support, can improve mobility and functional outcomes. However, patient responses vary depending on disease subtype and severity [8]. These strategies are particularly important in early and moderate disease stages, where functional preservation is a primary goal.

Surgical interventions, particularly total joint arthroplasty, are effective in advanced disease stages. However, factors such as implant lifespan, postoperative recovery, and patient suitability limit their broader use [9]. Overall, current management remains largely symptomatic, highlighting the unmet need for disease-modifying interventions.

Emerging mechanisms and therapeutic targets

Advances in molecular biology, biomechanics, and regenerative medicine have led to the exploration of new therapeutic approaches [6]. Research has identified several key mechanisms involved in OA progression, including chondrocyte (cartilage cell) senescence, ECM (the structural network surrounding cells) remodeling, synovial inflammation involving macrophage migration, and changes in subchondral bone structure [10]. These mechanisms provide a direct link between disease pathophysiology and potential therapeutic targets.

These findings have supported the development of regenerative therapies such as platelet-rich plasma (PRP), mesenchymal stem cell (MSC) therapies, exosome-based treatments, and tissue-engineered scaffolds [1]. In parallel, the development of disease-modifying osteoarthritis drugs (DMOADs) aims to target inflammatory pathways, reduce tissue breakdown, and promote tissue repair [11].

**Figure 1:**
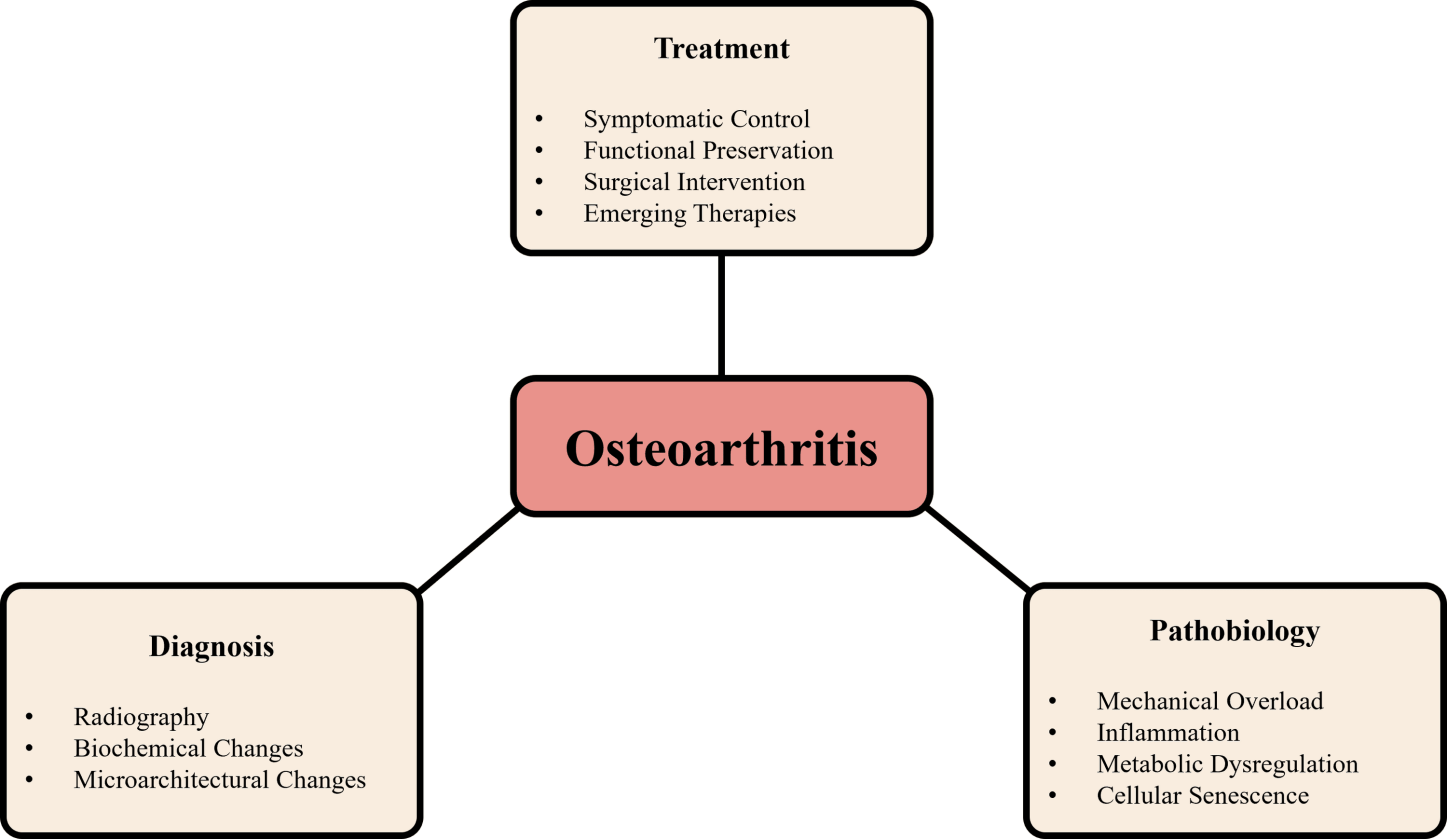
Overview of OA pathobiology, diagnosis, and treatment OA: osteoarthritis Created by authors using Microsoft PowerPoint (Microsoft Corporation, Redmond, WA, US).

However, clinical outcomes remain inconsistent. This is largely due to variability in patient responses, the absence of reliable early diagnostic biomarkers, and the lack of validated phenotype-based treatment strategies [12]. In other words, patients with OA do not all respond in the same way, making it difficult to apply a single standardized treatment approach. This variability underscores the importance of stratified or phenotype-based therapeutic approaches. Figure 1 shows the major components of OA.

Gaps and future directions

There are significant gaps between current diagnostic methods, treatment strategies, and long-term disease management. Existing classification systems have a limited ability to distinguish between biological subtypes of OA, restricting the ability to match individual patients with appropriate therapies [13]. Imaging techniques do not adequately capture dynamic biochemical changes within joint tissues. Additionally, available biomarkers lack sufficient validation for routine clinical use [13].

The long-term safety and effectiveness of regenerative therapies also remain uncertain, particularly in patients with different metabolic conditions, comorbidities, or mechanical loading patterns. Furthermore, the lack of integration between molecular findings and clinical decision-making remains a key barrier to effective translation.

These limitations highlight the need for more precise and individualized approaches to OA diagnosis and treatment. In particular, strategies that integrate molecular mechanisms with clinical features are required.

Aim and objectives of the review

In light of these gaps, this review aims to synthesize current evidence on OA pathophysiology, diagnostic approaches, and emerging therapeutic strategies, with a focus on linking molecular insights with clinically meaningful outcomes. The accumulation of translational evidence suggests that combined management strategies are necessary. These strategies should consider the interaction between joint tissues and systemic factors. Approaches that integrate metabolic regulation, biomechanical alignment (the distribution of forces across the joint), molecular targeting, and lifestyle modification may provide benefits beyond symptom control. Further progress in OA management requires the integration of scientific advances with clinically applicable models.

Methodology

Search Strategy

A structured literature search was conducted across major scientific databases, including PubMed, Scopus, and Web of Science, to identify relevant studies published between 2015 and 2026. The search strategy incorporated keywords related to OA pathophysiology, diagnostic approaches, conventional management, regenerative therapies, and emerging treatment strategies. Boolean operators and combinations of terms were used to improve search sensitivity and specificity. The search strategy included combinations such as (“osteoarthritis” AND “pathophysiology” OR “diagnosis” OR “treatment”), with database-specific adaptations applied where necessary. To ensure comprehensive coverage, reference lists of selected articles and relevant clinical guidelines were also manually screened. Abstracts and titles were initially reviewed to identify studies providing clinically relevant or mechanistic insights into OA.

Eligibility Criteria

Study selection was guided by predefined eligibility criteria. The inclusion criteria comprised original research articles, clinical trials, observational studies, systematic reviews, and meta-analyses addressing OA mechanisms, diagnosis, or management strategies within the specified timeframe. Preference was given to recent high-quality evidence, particularly randomized controlled trials, systematic reviews, meta-analyses, and updated clinical guidelines, to ensure relevance and methodological robustness.

The exclusion criteria included studies with poor methodological quality, articles not directly related to OA, non-human or veterinary studies without clear translational relevance, and studies focusing on outdated or non-clinically applicable interventions. Conference abstracts, editorials, and non-peer-reviewed sources were also excluded to maintain scientific rigor.

Study Selection Process

Following initial screening, full-text articles were assessed for eligibility and relevance. Study selection was performed through structured screening of titles, abstracts, and full texts, with discrepancies resolved through consensus. The selection process followed PRISMA-based reporting principles to enhance transparency and reproducibility.

Data Extraction

Data extraction focused on study design, population characteristics, interventions, outcomes, and key mechanistic findings related to OA. Studies were prioritized based on clinical applicability, strength of evidence, and contribution to advancing understanding of OA.

Quality Assessment

The methodological quality of included studies was appraised using established critical appraisal frameworks appropriate to study design, including tools aligned with Cochrane risk-of-bias principles and A Measurement Tool to Assess Systematic Reviews 2 (AMSTAR-2) recommendations for systematic reviews.

Data Synthesis

A narrative synthesis approach was used to integrate findings across heterogeneous study designs, with emphasis on linking molecular mechanisms to clinical outcomes and therapeutic strategies.

## Review

Epidemiology and risk factors of osteoarthritis

OA is one of the most common chronic joint conditions worldwide, with prevalence increasing significantly in ageing populations [13]. Incidence rates rise markedly after middle age, highlighting age as a primary determinant of disease onset. Sex-specific patterns indicate a higher incidence in females, particularly after menopause, suggesting a role of hormonal changes in maintaining joint tissue integrity [14]. Epidemiological studies also demonstrate geographic and ethnic variability, which may reflect differences in genetic predisposition, lifestyle factors, and access to healthcare services [4]. The increasing burden of OA contributes to chronic pain, functional decline, and long-term disability, thereby imposing substantial clinical and socioeconomic challenges [1].

Several risk factors contribute to the development and progression of OA, involving complex interactions between biological, metabolic, genetic, and environmental pathways [15]. These risk factors can be broadly categorized into intrinsic (age, genetics, and sex) and extrinsic (mechanical, lifestyle, and environmental) factors, which together influence disease onset and progression. Age-related changes play a central role in joint degeneration. These include reduced ECM turnover, increased cellular senescence, and diminished regenerative capacity of joint tissues [16]. Genetic susceptibility further influences cartilage structure, inflammatory responses, and subchondral bone remodeling, with multiple susceptibility loci identified in genomic studies [2]. Metabolic and systemic factors also contribute significantly. Obesity and metabolic syndrome promote chronic low-grade inflammation and mechanical overload, accelerating cartilage degradation and subchondral bone changes [17]. Elevated levels of adipokines further enhance catabolic processes within joint tissues [18].

Mechanical and environmental factors are equally important. Repetitive joint loading through occupational activities such as kneeling, squatting, and heavy lifting can induce early degenerative changes [19]. Similarly, high-impact sports and prior joint injuries disrupt normal biomechanics, leading to joint instability and increased susceptibility to OA [14]. Conversely, physical inactivity and reduced muscle strength diminish joint support, increasing mechanical stress during routine activities [7]. Collectively, these interacting factors contribute to heterogeneous disease phenotypes, influencing the onset, severity, and progression of OA across different patient populations [20].

Advances in understanding osteoarthritis pathophysiology

Modern understanding of OA characterizes it as a complex joint disorder driven by coordinated biological, metabolic, and biomechanical disturbances across multiple tissues [18]. Earlier concepts that focused solely on cartilage degeneration have been replaced by a whole-joint perspective, where structural, cellular, and biochemical changes occur simultaneously within the joint environment [21]. This integrated model incorporates contributions from synovial inflammation, subchondral bone remodeling, meniscal degeneration, and matrix degradation mediated by inflammatory and enzymatic pathways [22]. At a structural level, articular cartilage degeneration remains a central feature of OA. This process is initiated by disruption of chondrocyte homeostasis, increased oxidative stress, and reduced anabolic signaling [23]. Catabolic enzymes, including matrix metalloproteinases (MMPs) and aggrecanases, accelerate ECM degradation, leading to reduced load-bearing capacity of cartilage [15]. These structural changes are further exacerbated by biomechanical stress, which impairs normal cellular mechanotransduction and accelerates tissue deterioration [24].

At a molecular and inflammatory level, synovial tissue plays a critical role in disease progression. Synovial inflammation is characterized by macrophage infiltration, angiogenesis, and increased secretion of pro-inflammatory cytokines, which contribute to persistent joint inflammation and nociceptive sensitization [21]. Cytokines such as interleukin-1β and tumor necrosis factor activate signaling cascades that increase MMP expression, inhibit collagen synthesis, and amplify inflammatory responses within the joint [18]. Subchondral bone remodeling represents another key pathophysiological axis. It is characterized by increased bone turnover, microarchitectural changes, and osteophyte formation [25]. Altered communication between subchondral bone and cartilage (bone-cartilage crosstalk) further disrupts chondrocyte function and facilitates the transfer of inflammatory mediators across the osteochondral interface [26]. Meniscal degeneration also contributes to disease progression by increasing joint instability and redistributing mechanical load, thereby promoting focal cartilage damage [27].

In addition to structural and inflammatory mechanisms, metabolic and cellular processes have emerged as important contributors to OA pathophysiology. Adipokines and advanced glycation end products disrupt ECM integrity and increase oxidative stress within joint tissues [28]. Emerging evidence also highlights the roles of cellular senescence, mitochondrial dysfunction, and epigenetic modifications in sustaining chronic disease progression [29]. These interacting mechanisms create a self-perpetuating cycle of joint damage, inflammation, and pain. Importantly, this integrated understanding provides a direct rationale for targeted therapeutic strategies, including anti-inflammatory agents, regenerative therapies, and interventions aimed at modifying metabolic and biomechanical risk factors. The overall evidence supports the concept that OA is a multifactorial disease requiring a comprehensive biological and clinical approach for effective management [15]. Table 1 shows significant pathophysiological elements of OA and their molecular and clinical characteristics.

**Table 1:** Key pathophysiological components in OA MMPs: matrix metalloproteinases; IL-1β: interleukin-1 beta; TNF-α: tumor necrosis factor alpha; RANKL: receptor activator of nuclear factor kappa-B ligand; OA: osteoarthritis; ECM: extracellular matrix

Pathophysiological element	Central mechanisms	Clinical implications	Molecular drivers	References
Cartilage degradation	ECM breakdown; chondrocyte dysfunction	Reduced load-bearing capacity; progressive joint dysfunction	MMPs, aggrecanases, oxidative stress	[15]
Synovial inflammation	Macrophage activation; cytokine-mediated inflammation	Pain sensitization, joint swelling, and effusion	IL-1β, TNF-α, and inflammatory mediators	[21]
Subchondral bone remodeling	Increased bone turnover, sclerosis, and microarchitectural changes	Altered load distribution; osteophyte formation	RANKL, Wnt signaling pathways	[22,23]
Meniscal degeneration	Structural disruption; loss of meniscal integrity	Joint instability; increased focal cartilage stress	Matrix degradation, inflammatory mediators	[27]
Biomechanical stress pathways	Impaired mechanotransduction; abnormal load transmission	Focal cartilage damage; accelerated disease progression	Integrins, ion-channel signaling	[24]

Clinical presentation and natural course of disease

OA is characterized by a cluster of symptoms that develop gradually over time. Early manifestations typically include intermittent joint pain triggered by mechanical stress or prolonged activity [30]. Pain commonly affects weight-bearing joints such as the knee, hip, and spine, and typically worsens with movement while improving with rest [31]. Joint stiffness frequently occurs after periods of inactivity, particularly in the morning, and usually resolves within a short duration with continued movement [32].

As the disease progresses, patients may develop a reduced range of motion, crepitus, joint enlargement, and functional impairment, reflecting ongoing structural deterioration [33]. These clinical features correspond to cumulative pathological changes involving cartilage degradation, synovial inflammation, subchondral bone alterations, and periarticular tissue involvement [23]. In established stages, radiographic findings commonly include joint space narrowing, osteophyte formation, subchondral sclerosis, and cystic changes [34]. MRI provides enhanced visualization of early structural abnormalities, including cartilage defects, bone marrow lesions, meniscal degeneration, and synovial inflammation. This enables earlier detection of disease activity before radiographic changes become evident [35]. However, symptom severity does not always correlate with structural findings, reflecting a complex interplay between nociceptive pathways, inflammatory mediators, and biomechanical stress [30].

The natural course of OA is heterogeneous and varies between individuals. Disease progression is influenced by interacting metabolic, biomechanical, and inflammatory factors [28]. An inflammatory phenotype is characterized by episodic pain, joint effusion, and increased synovial activity [21]. A metabolic phenotype is associated with obesity, dyslipidemia, and systemic inflammation, contributing to accelerated cartilage degradation [24]. A biomechanical phenotype is linked to malalignment, ligamentous instability, or focal overload, often following prior joint injury [27]. These phenotypic variations demonstrate that OA progression is not uniform, and they highlight the importance of phenotype-based approaches for understanding disease progression and guiding targeted management strategies [22].

Diagnostic approaches: clinical, radiologic, and biomarker-based tools

The diagnostic assessment of OA involves a combination of clinical evaluation, imaging techniques, and emerging biomarker-based approaches to identify disease presence, stage, and progression [36]. An integrated diagnostic framework is essential, as no single modality sufficiently captures the full spectrum of structural and molecular changes in OA.

Clinical assessment

Clinical diagnosis typically begins with evaluation of symptom patterns, including joint pain characteristics, duration of stiffness, joint tenderness, crepitus, and functional limitation [37]. Pain is usually activity-related and localized to affected joints, while stiffness is often transient and occurs after periods of inactivity. These clinical features provide indirect insight into structural compromise and synovial involvement [21]. Physical examination further contributes to assessment by evaluating joint alignment, gait abnormalities, ligamentous stability, and range-of-motion deficits [24]. These findings help differentiate mechanical dysfunction from inflammatory components and guide further diagnostic evaluation.

Radiologic imaging

Radiologic imaging plays a central role in confirming structural joint changes [33]. Conventional radiography remains the most widely used modality due to its accessibility and standardized diagnostic criteria. Typical findings include joint space narrowing, osteophyte formation, subchondral sclerosis, and cystic changes [34]. However, radiographs have limited sensitivity for early disease and often detect only advanced structural abnormalities [35]. MRI provides greater sensitivity for early and subtle changes, including cartilage defects, bone marrow lesions, meniscal abnormalities, synovial thickening, and joint effusion. This makes MRI particularly valuable for early detection and for assessing disease activity before radiographic changes become evident. Ultrasound offers a complementary imaging modality by enabling dynamic assessment of synovitis, joint effusion, osteophyte margins, and soft tissue abnormalities [38]. Its real-time capability and accessibility make it useful in clinical settings for monitoring inflammatory activity and guiding interventions.

Biomarker-based approaches

Emerging molecular biomarkers provide the potential to detect early biochemical and metabolic alterations before structural damage becomes apparent [36]. Biomarkers derived from serum, synovial fluid, and urine are being investigated to reflect cartilage degradation, subchondral bone remodeling, inflammatory activity, and metabolic dysregulation [37]. Specific markers, such as cartilage oligomeric matrix protein (COMP), C-terminal telopeptides, and pro-inflammatory mediators, have shown potential in identifying high-risk disease phenotypes and monitoring therapeutic responses [39]. However, despite promising findings, the lack of standardized and validated biomarkers currently limits their routine clinical application [13].

Integrated diagnostic approach

Combining clinical assessment, imaging modalities, and biomarker analysis offers a more comprehensive approach to OA diagnosis and disease characterization. This integrated strategy improves diagnostic accuracy, enables earlier detection, and supports the development of personalized treatment strategies [33]. Table 2 provides the important diagnostic instruments of OA and their related strengths and clinical importance.

**Table 2:** Diagnostic modalities used in OA ROM: range of motion; MRI: magnetic resonance imaging; COMP: cartilage oligomeric matrix protein; CTX-II: C-terminal telopeptide of type II collagen; OA: osteoarthritis

Diagnostic category	Principal features	Clinical utility	Key indicators/technologies	References
Clinical assessment	Pain characteristics; joint tenderness; crepitus; stiffness	Symptom evaluation; functional assessment	ROM testing; gait analysis	[37]
Radiography	Joint space narrowing; osteophytes; subchondral sclerosis	Structural assessment; disease severity grading	Kellgren-Lawrence grading system	[34]
MRI	Cartilage defects; bone marrow lesions; synovitis	Early detection; comprehensive joint evaluation	T2 mapping; cartilage thickness analysis; synovial scoring	[33]
Ultrasound	Synovitis; joint effusion; osteophyte margins	Dynamic soft-tissue assessment; inflammation monitoring	Power Doppler imaging	[38]
Biomarker analysis	Cartilage degradation markers; inflammatory mediators	Early detection; disease monitoring; phenotype stratification	COMP; CTX-II; cytokine profiling	[16]

Traditional pharmacologic management of osteoarthritis

Traditional pharmacologic management of OA is primarily focused on symptom relief, preservation of joint function, and reduction of inflammation [40]. These approaches are central to clinical care but do not address the underlying disease mechanisms. NSAIDs are the most commonly used agents for managing OA-related pain. They reduce prostaglandin-mediated inflammation and are effective in improving mobility and daily functional activities [41]. Oral NSAIDs provide systemic effects, whereas topical formulations offer localized action with reduced systemic exposure, making them preferable in certain patient populations [42]. However, their use is limited by potential adverse effects, including gastrointestinal complications, renal impairment, and cardiovascular risks, particularly in individuals [32].

Analgesics such as acetaminophen remain an option for patients with mild pain or those who cannot tolerate NSAIDs [43]. Their analgesic efficacy is generally modest, and prolonged use at higher doses may be associated with hepatotoxicity [44]. Opioid medications are reserved for select cases with severe pain unresponsive to other therapies. Their use is restricted due to concerns related to tolerance, dependence, and adverse effects, limiting their role in long-term management [40].

Intra-articular corticosteroid injections are commonly used for short-term symptom relief, particularly during disease flares. These injections exert anti-inflammatory effects by reducing synovial inflammation and cytokine activity, leading to temporary improvement in pain and joint swelling [32]. However, repeated administration may adversely affect cartilage integrity and joint homeostasis [45]. Hyaluronic acid injections are intended to restore the viscoelastic properties of synovial fluid, thereby improving joint lubrication and shock absorption [46]. Clinical outcomes are variable, with better responses typically observed in early-stage disease and in patients with lower levels of inflammation [40].

**Figure 2:**
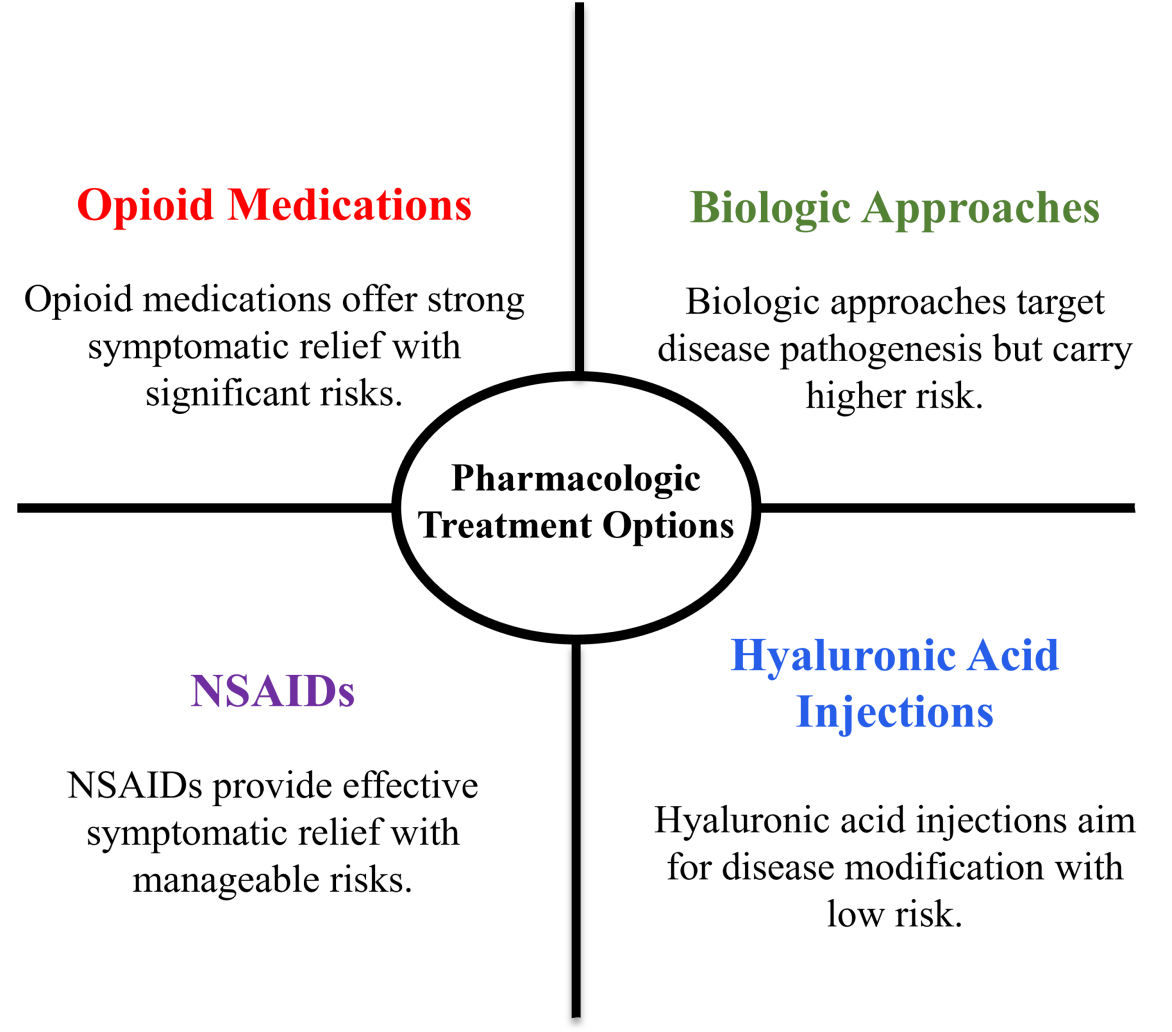
Core therapeutic options for OA management NSAIDs: nonsteroidal anti-inflammatory drugs; OA: osteoarthritis Created by authors using Microsoft PowerPoint (Microsoft Corporation, Redmond, WA, US).

Overall, while traditional pharmacologic therapies are effective in symptom control, they do not modify disease progression. This limitation underscores the need for adjunctive non-pharmacologic strategies and the development of disease-modifying and biologic therapies targeting underlying pathophysiological mechanisms [47]. The principal treatment strategies for OA are outlined in Figure 2.

Non-pharmacologic interventions and lifestyle modification

Non-pharmacologic approaches play a central role in the management of OA, focusing on non-surgical strategies that reduce symptoms and improve functional capacity [48]. These interventions form the foundation of long-term management and are recommended across all disease stages. Weight loss is a key intervention for individuals who are overweight or obese. Reduction in body weight decreases mechanical load on weight-bearing joints and improves functional outcomes, although uncertainty remains regarding the optimal dietary approach and the extent of weight reduction required for maximal benefit [49]. Even modest weight loss has been shown to improve mobility and daily functional performance [49].

Exercise therapy is a cornerstone of OA management. Structured exercise programs improve periarticular muscle strength, joint stability, and proprioception [50]. Aerobic conditioning, resistance training, and flexibility exercises have demonstrated benefits in reducing pain, improving gait mechanics, and enhancing overall physical capacity [51]. Aquatic therapy provides an alternative for patients with limited weight-bearing tolerance, allowing movement with reduced mechanical stress on joints [52]. Physiotherapy supports individualized rehabilitation through manual therapy, neuromuscular re-education, and targeted strengthening programs [20]. These interventions address biomechanical deficits, improve joint alignment, and enhance functional mobility [24]. Orthotic devices, including valgus braces, lateral wedge insoles, and supportive footwear, help redistribute joint loading and reduce focal stress, particularly in patients with malalignment or compartmental disease [17].

Patient education is an essential component of long-term disease management. It promotes self-management, activity modification, and the adoption of joint-protective strategies, thereby improving adherence and clinical outcomes [25]. Digital health technologies, such as tele-rehabilitation platforms, wearable motion sensors, and self-management applications, further enhance accessibility to personalized care and enable continuous monitoring of functional progress [23]. Collectively, non-pharmacologic interventions provide consistent benefits across different OA phenotypes, contributing to symptom control, functional preservation, and improved quality of life. These strategies also complement pharmacologic and procedural treatments by targeting biomechanical and behavioral factors that influence disease progression [18].

Surgical options and indications

Surgical intervention in OA is considered when conservative management fails to provide adequate symptom relief, functional improvement, or joint stability [32]. The choice of procedure depends on disease severity, anatomical involvement, alignment status, and patient-specific functional demands. Joint-preserving procedures may be appropriate in early to intermediate disease stages, whereas advanced degeneration often necessitates reconstructive interventions [20]. Arthroscopic procedures, including debridement, lavage, and removal of loose bodies, aim to relieve mechanical symptoms. However, evidence suggests limited long-term benefit in degenerative OA, with potential short-term improvement only in selected cases with mechanical locking or focal meniscal pathology [32].

Corrective osteotomy procedures, such as high tibial osteotomy and distal femoral osteotomy, are used to address malalignment and redistribute load across the joint [31]. These techniques are particularly beneficial in unicompartmental knee OA, especially in younger and more active patients with varus or valgus deformities [32]. Unicompartmental arthroplasty is indicated when the disease is confined to a single compartment. This approach preserves native ligament structures and joint kinematics, enabling faster recovery and improved functional outcomes in appropriately selected patients [17]. Total joint arthroplasty remains the definitive treatment for end-stage OA, providing substantial pain relief and restoration of functional mobility [32].

Advancements in implant design and surgical techniques have improved long-term outcomes and reduced revision rates. However, complications such as infection, implant loosening, and periprosthetic fractures remain important considerations [19]. Postoperative outcomes are influenced by factors including preoperative alignment, muscle strength, metabolic status, and adherence to rehabilitation protocols [20]. Enhanced recovery protocols have further contributed to reduced perioperative morbidity and faster functional recovery. Emerging innovations, including robotic-assisted surgery, patient-specific implants, and minimally invasive techniques, continue to refine surgical outcomes and extend durability across different OA phenotypes [34]. Table 3 shows the main surgical interventions in the treatment of OA and their usage in clinical practice.

**Table 3:** Overview of surgical interventions in OA OA: Osteoarthritis

Surgical approach	Primary objective	Ideal candidate profile	Key advantages/limitations	References
Arthroscopy	Debridement; removal of loose bodies	Mechanical locking; focal intra-articular pathology	Short-term symptom relief; limited long-term benefit in degenerative OA	[32]
High tibial/distal femoral osteotomy	Alignment correction; load redistribution	Unicompartmental OA with varus or valgus malalignment; younger, active patients	Joint preservation; delays need for arthroplasty; prolonged rehabilitation	[32]
Unicompartmental arthroplasty	Partial joint replacement	Isolated compartment OA with intact ligaments	Faster recovery; preservation of native kinematics; risk of disease progression in other compartments	[17]
Total joint arthroplasty	Complete joint replacement	End-stage OA with severe pain and functional limitation	Significant pain relief; durable outcomes; risk of infection, loosening, and prosthetic complications	[32]
Minimally invasive/robotic-assisted techniques	Precision implant placement; alignment optimization	Selected patients across OA severity depending on surgical indication	Improved accuracy and alignment; higher cost and limited availability	[34]

Emerging biological and regenerative therapies

Emerging biological and regenerative therapies are being explored as potential approaches to modify OA progression by targeting cellular, molecular, and structural components of the joint environment [40]. These strategies aim to extend beyond symptom control by influencing tissue repair processes and disease-related pathways. PRP therapy delivers a concentrated source of growth factors that may stimulate chondrocyte activity, support ECM synthesis, and modulate local inflammatory responses [53]. Clinical studies have reported improvements in pain and joint function, particularly in early to moderate disease stages. However, the overall evidence remains heterogeneous, and variability in preparation techniques, platelet concentration, and activation protocols contributes to inconsistent findings across studies [8,9].

MSC therapies represent another regenerative approach due to their multipotent differentiation capacity and immunomodulatory effects [53]. MSCs derived from bone marrow, adipose tissue, or umbilical cord have been associated with cartilage repair through paracrine signaling, ECM production, and modulation of synovial inflammation [11]. Although some studies report improvements in structural and symptomatic outcomes, interpretation is limited by small sample sizes, methodological variability, and insufficient long-term safety and efficacy data [12]. Exosome-based therapies extend aspects of MSC-mediated effects by delivering bioactive molecules, including microRNAs, cytokines, and growth factors, within extracellular vesicles. These approaches may enable targeted modulation of pathways involved in cartilage degradation and inflammation while avoiding some risks associated with cell-based therapies [18]. However, exosome-based interventions remain in early investigational stages, with limited clinical validation and evolving regulatory considerations.

Tissue engineering strategies aim to enhance regenerative potential through biomaterial scaffolds that support chondrocyte attachment, ECM formation, and mechanical stability [28]. Biomaterials such as collagen, hyaluronic acid, and synthetic polymers are used to facilitate guided tissue regeneration. Advances in bioprinting technologies have enabled the development of anatomically tailored constructs designed to approximate native cartilage architecture and biomechanical properties [27]. Despite these developments, challenges related to integration with native tissue, durability, scalability, and cost remain significant barriers to clinical translation. Overall, while regenerative therapies represent a promising area of investigation, their current application is limited by heterogeneity in study design, lack of standardized protocols, and insufficient long-term outcome data. Further large-scale, well-designed clinical studies are required to clarify their safety, consistency, and clinical role within phenotype-specific OA management strategies.

Development of disease-modifying osteoarthritis drugs

DMOADs focus on slowing or altering structural joint degeneration by targeting key molecular pathways involved in cartilage degradation, synovial inflammation, and subchondral bone remodeling [19]. Enzyme-targeting strategies include inhibitors of MMPs and aggrecanases, which aim to reduce cartilage degradation by limiting enzymatic breakdown of the ECM [15]. Although these approaches are mechanistically promising, their clinical application remains limited due to challenges related to selectivity, potential off-target effects, and concerns regarding long-term safety.

Modulation of subchondral bone remodeling represents another therapeutic target. RANKL inhibitors and Wnt signaling modulators are being investigated for their potential to stabilize bone architecture and support osteochondral homeostasis [22]. Anabolic agents such as fibroblast growth factor-18 (FGF-18) analogues have been associated with increases in cartilage thickness in some studies, although translation of these structural findings into consistent clinical benefit remains uncertain [39].

Emerging therapeutic approaches include senolytic agents targeting senescent cells, antioxidants aimed at mitigating mitochondrial dysfunction, and epigenetic modulators influencing gene expression related to OA progression [29]. These strategies reflect advances in understanding disease biology but remain largely in early-phase investigation with limited clinical validation.

Overall, the development of DMOADs is constrained by disease heterogeneity, lack of reliable biomarkers for patient stratification, and challenges in demonstrating sustained structural and symptomatic outcomes in clinical trials. Integration of imaging biomarkers and molecular profiling may improve prediction of treatment response and support the development of more targeted therapeutic strategies [36].

Unresolved challenges in osteoarthritis research and clinical care

OA remains associated with several unresolved challenges that limit progress in diagnosis, treatment, and long-term disease management [18]. A major challenge is the marked heterogeneity in clinical presentation, driven by complex interactions between metabolic, biomechanical, and inflammatory factors [28]. This variability complicates phenotypic classification and contributes to inconsistent responses to treatment, thereby limiting the effectiveness of uniform therapeutic approaches [33].

Diagnostic limitations persist, as conventional radiography primarily detects structural changes at relatively advanced stages of disease [34]. Early biochemical and molecular alterations in cartilage, synovium, and subchondral bone are often not captured using current diagnostic tools, which restricts opportunities for early intervention [39]. Although biomarkers have been investigated for early detection and disease stratification, their clinical application remains limited by a lack of standardized thresholds, inconsistent validation, and variability across populations [37].

Therapeutic challenges also remain substantial. Current pharmacologic and procedural interventions primarily provide symptomatic relief without consistently modifying long-term structural progression [31]. Regenerative and biologic therapies have shown potential in some studies but demonstrate variable outcomes, reflecting differences in methodology, patient selection, and disease phenotype [8]. Uncertainty regarding long-term safety, durability, and optimal dosing further limits their broader clinical implementation [46].

The complex interplay between cartilage degradation, synovial inflammation, and subchondral bone remodeling remains incompletely characterized [25]. This limits the development of therapies capable of targeting multiple interconnected disease pathways simultaneously [15]. Surgical outcomes also vary depending on factors such as alignment, muscle strength, comorbidities, and adherence to rehabilitation, contributing to variability in long-term prognosis [20]. These challenges highlight the need for improved diagnostic precision, more robust phenotypic stratification, and integration of molecular, imaging, and clinical data to support more targeted and consistent management strategies in OA.

Limitations and future recommendations

This review synthesizes evidence from diverse study designs, which introduces variability in methodological quality, diagnostic criteria, and reported outcomes across included studies. Heterogeneity in study populations, intervention protocols, and follow-up durations limits direct comparability and reduces the generalizability of findings. Limited availability of validated early diagnostic tools, particularly biomarkers and advanced imaging modalities, further constrains comprehensive assessment of disease onset and progression. Additionally, inconsistencies in reporting standards and outcome measures across studies hinder robust evaluation of therapeutic efficacy. Regenerative and biologic therapies present further limitations due to variability in preparation techniques, dosing strategies, and patient selection criteria. These inconsistencies complicate the interpretation of clinical outcomes and limit reproducibility across different settings.

Future research should prioritize the development of standardized diagnostic frameworks integrating molecular biomarkers, imaging findings, and clinical parameters to enable earlier and more accurate detection of OA. Validation of reliable biomarkers for phenotype-based stratification will be essential to support precision medicine approaches. Large-scale, longitudinal studies with uniform outcome measures are required to assess long-term structural and functional benefits of emerging pharmacologic and regenerative therapies.

Further efforts should also focus on refining patient selection criteria, standardizing treatment protocols, and integrating multimodal data to guide personalized therapeutic decision-making. Advancements in surgical techniques, postoperative rehabilitation strategies, and patient-reported outcome measures should be aligned with these developments to improve overall clinical effectiveness. A coordinated, multidisciplinary approach that bridges molecular research, clinical practice, and technological innovation will be critical for advancing OA management and improving long-term patient outcomes.

## Conclusions

OA is a complex and multidimensional joint disorder driven by interactions between mechanical loading, inflammatory processes, metabolic factors, and progressive structural changes across cartilage, synovium, subchondral bone, and periarticular tissues. Contemporary understanding characterizes OA as a whole-joint disease rather than an isolated cartilage pathology, with important implications for diagnosis and management. Advances in imaging and emerging biomarker strategies have improved disease characterization; however, early detection remains limited by a lack of standardization, incomplete validation, and variable clinical applicability. Current pharmacologic and non-pharmacologic approaches provide symptomatic relief and functional improvement but do not consistently alter long-term structural progression. Surgical interventions offer clinical benefit in advanced disease, supported by ongoing refinements in alignment techniques, implant design, and perioperative care. Emerging biological and regenerative therapies are being investigated for their potential to target underlying disease mechanisms, including matrix repair and modulation of cellular signaling, although clinical outcomes remain variable and require further validation. Overall, despite notable advances, important gaps persist in early diagnosis, disease modification, and personalized management, highlighting the need for integrated diagnostic frameworks and continued evaluation of targeted therapeutic strategies across diverse OA phenotypes.
